# Preparation of aspirin inhalable powder by ultrasound-intensified anti-solvent crystallization for pulmonary drug delivery

**DOI:** 10.1016/j.ultsonch.2025.107464

**Published:** 2025-07-11

**Authors:** Yan Zhao, Kai Feng, Boxin Liu, Zhihao Zhang, Haozhou Huang, Mateng Chen, Qingzhen Zhang, Gang Yang, Mengxing Lin, Yulong Zhang, Hanhan Li, Ning Xue, Kaiqi Shi, Qiang Da, Bin Dong

**Affiliations:** a The First Affiliated Hospital of Nanjing Medical University, 300 Guangzhou Road, Nanjing 210029, China; b Department of Pharmaceutical Engineering, China Pharmaceutical University, Nanjing 210009, China; c State Key Laboratory of Natural Medicines, Key Laboratory of Drug Quality Control and Pharmacovigilance, School of Pharmacy, China Pharmaceutical University, Nanjing 210009, China; d Courant Institute of Mathematical Sciences, New York University, USA; e Suzhou Inhal Pharma Co., Ltd, Suzhou, Jiangsu 215000, China; f Eastern Institute for Advanced Study, Eastern Institute of Technology, Ningbo, China; g National Heart & Lung Institute, Faculty of Medicine, Imperial College London, Guy Scadding Building, Cale Street, London SW3 6LY, United Kingdom; h Department of Computer Science, University of Nottingham Ningbo China, Ningbo, Zhejiang 31500, China; i Engineering Research Center for Smart Pharmaceutical Manufacturing Technologies, Ministry of Education, China Pharmaceutical University, Nanjing 210009, China

**Keywords:** Aspirin, Inhalable powder, Ultrasound-intensified antisolvent crystallization, Machine learning, Pulmonary drug delivery

## Abstract

Aspirin is an antiplatelet agglutinating drug used clinically for the prevention and treatment of angina pectoris, myocardial infarction, and cerebral thrombosis. In this study, aspirin inhalable powder was prepared by ultrasound-intensified anti-solvent crystallization (UIAC) and developed for rapid antiplatelet aggregation, which could reduce the dose and gastrointestinal irritation. The particle size distribution, morphology, density, fluidity, and in vitro aerodynamic performance of the as-prepared powders were systematically evaluated. Meanwhile, machine learning methodology, specifically utilizing the Decision Tree Regressor in conjunction with Shapley Value analysis, was applied to elucidate the influence of critical process parameters within the production process. The powder flowability could be improved by the addition of excipients L150 and L-leucine (Leu). The value of fine particle fraction (FPF) increased from 10.40 % to 45.86 % when adding L150 (60 %, w/w) and Leu (5 %, w/w). The cytotoxicity study of aspirin inhalable powder was performed at cellular level, and demonstrated aspirin powder had no significant toxic effect. The Calu-3 cell monolayer interfaced to simulate lung epithelial tissue, demonstrated the high permeability of inhalable powder in lung. Pharmacokinetics were investigated in healthy rats, compared with oral administration, the T_max_ of inhale administration (10 min) was significantly shorter than oral administration (30 min), and the AUC was 1.91 times higher than that of the oral administration, demonstrating that pulmonary drug delivery accelerated the absorption and increased the bioavailability of aspirin.

## Graphical abstract

**Figure:**
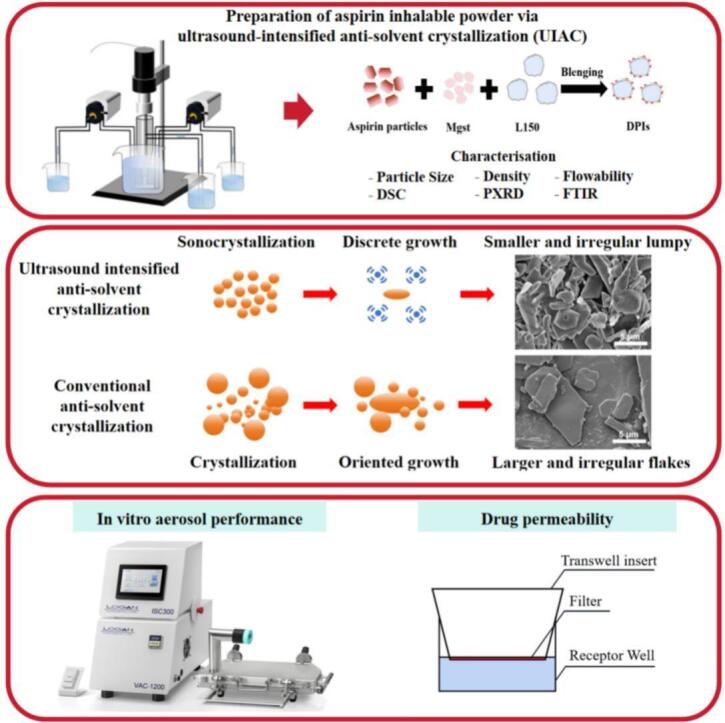

## Nomenclature

DPIs: Dry powder inhalations
ASA: Aspirin
API: Active pharmaceutical ingredient
SFD: Spray freeze drying
SEM: Scanning electron microscope
HPLC: High performance liquid chromatography
DSC: Differential scanning calorimetry
XRD: X-ray diffractometer
FTIR: Fourier transform infrared analysis
CI: Carr’s index
HR: Hausner ratio
ρ_B_: Bulk density
ρ_T_: Tap density
Leu: L-Leucine
L150: Lactose Monohydrate
PBS: Phosphate buffered saline
GMB: Gamble’s solution
ALF: Artificial lysosomal fluid
NGI: Next generation impactor
ED: Emitted dose
FPF: Fine particle fraction
MMAD: Mass median aerodynamic diameter
GSD: Geometric standard deviation
RH: Relative humidity
MTT: 3-(4,5-dimethyl-2-thiazolyl)-2,5-diphenyl-2-H-tetrazolium bromide
HBSS: Hank's balanced salt solution
SA: Salicylic acid
AA: Arachidonic acid
ADP: Adenosine 5′-diphosphate
PA: Platelet aggregation

## 1 Introduction

As a classic non-steroidal anti-inflammatory drug for antipyretic and analgesic, aspirin (known as acetylsalicylic acid, ASA) has been widely used for the treatment of pain, fever, and inflammation (including rheumatoid arthritis, osteoarthritis, etc.) [1]. In addition, as an anti-platelet drug, aspirin is also clinically used for the treatment and prevention of angina pectoris, myocardial infarction, and cerebral thrombosis due to its anti-thrombotic effect in vivo [2]. Aspirin could inhibit the formation of thromboxane A2 (TXA2) by irreversible acetylation platelet cyclooxygenase, mainly acetylating the serine 530 site of cyclooxygenase-1 (COX-1), resulting in the antiplatelet aggregation and antithrombotic effects [[3], [4], [5]].

However, it has been demonstrated that aspirin could cause peptic ulcers, and increase the risk of gastrointestinal bleeding, peptic ulcer, and other gastroduodenal mucosal injuries [6,7]. A study called the “UGLA Survey” showed that the most shoudagastroesophageal reflux, followed by heartburn and acid reflux [[8], [9], [10]]. Different aspirin formulations have been developed to elevate these adverse effects, including buffered aspirin and enteric-coated (EC) aspirin [[11], [12], [13]]. However, endoscopic studies have shown that using of EC aspirin tablets could reduce gastric mucosal lesions compared with regular aspirin tablets, but small intestinal mucosal lesions become more severe, so EC aspirin tablets did not significantly alter the incidence of gastrointestinal complications and bleeding clinically induced by aspirin. Buffered aspirin could reduce the incidence of gastric mucosal erosion, but could not reduce the incidence of peptic ulcers [14].

Pulmonary drug delivery (PDD) is a clinical treatment for various lung diseases and systemic diseases [15,16]. The drug could be delivered directly to the lung through nebulization, absorbed into the systemic circulation, and metabolized by bypassing the gastrointestinal tract and the liver, resulting in rapid pharmacological action [[17], [18], [19]]. As a new type of PDD form, dry powder inhalations (DPIs) could be stored in capsules, dispersed by special inhalation devices, actively inhaled by the patient and entered into lungs through the airflow, and exhibited many advantages [[20], [21], [22]]. For the PDD system, the particle size of DPIs affects the efficiency of pulmonary deposition significantly. The particles which greater than 5 μm are easily deposited in the upper respiratory tract or main bronchial tubes and exhibit poor inhalation performance. However, the particles smaller than 1 μm are exhaled with respiration. Generally, inhalable powder should have suitable particle sizes (1–5 μm) for efficient lung delivery and deposition [23,24].

Anti-solvent crystallization method has been widely used to prepare micronized particles [25]. Besides, ultrasound can facilitate crystallization by controlling the nucleation process, shortening the induction time and substable interval [26]. Ultrasound could enhance the mixing effect between anti-solvent and solvent, and rapidly generate high levels of supersaturation and induces higher nucleation rates [27]. Zhong et al. evaluated the effect of ultrasound on the kinetics of antisolvent sucrose crystallization [28]. The results showed that ultrasound significantly increased nucleation rate and reduced average crystal size. Thus, the average size of sucrose particles was reduced from 133.8 µm to 80.5 µm, while the nucleation rate was increased from 4.87 × 10^9^ m^−3^ s^−1^ to 1.18 × 10^11^ m^−3^ s^−1^ when applying ultrasound. Furthermore, ultrasound could produce cavitation bubbles in the gas–liquid interface and increase the nucleation rate [29]. Shock waves generated by cavitation bubbles could form secondary nucleation (heterogeneous nucleation) sites, leading to an increase of small crystals. The nucleation energy barrier around the cavitation bubbles is dramatically decreased, leading to the reduction of crystals bonding force and prevention of crystal agglomeration. Meanwhile, the thickness of the diffusion layer near the surface of the crystal could be reduced by the turbulent flow, and produces high-speed collisions among crystal molecules, leading to further raising crystals amount and suppressing particle growth [30,31].

In this work, ultrasound-intensified anti-solvent crystallization (UIAC) technology was developed to prepare aspirin inhalable powders. The UIAC technology can rapidly enhance the mixing intensity between solvent and anti-solvent, generate higher supersaturation and nucleation rate, results in the generation of uniform aspirin suspension and smaller crystals through the “bottom-up” method [32]. Besides, the utilization of the decision tree approach and Shapley value analysis serves as tools to visualize and interpret experimental outcomes, providing insight into how experimental conditions impact the results. Moreover, pulmonary delivery of aspirin inhalable powders could bypass gastrointestinal and hepatic metabolism, and rapidly enter the bloodstream through the pulmonary circulation, enabling rapid absorption and improved bioavailability of aspirin.

## 2 Experimental

### 2.1 Materials

Aspirin (Shandong Xinhua Pharmaceutical Co., Ltd., 99 %), Lactose Monohydrate L150 (Inhalac®, Meidenraum, Germany), L-Leucine (Aladdin, Shanghai Jingpure Biochemical Science and Technology Co., Ltd.), Ethanol (Greagent, Shanghai Taitan Science and Technology Co., Ltd.).

### 2.2 Preparation process

#### 2.2.1 Preparation of aspirin inhalable powders

**Fig. 1:**
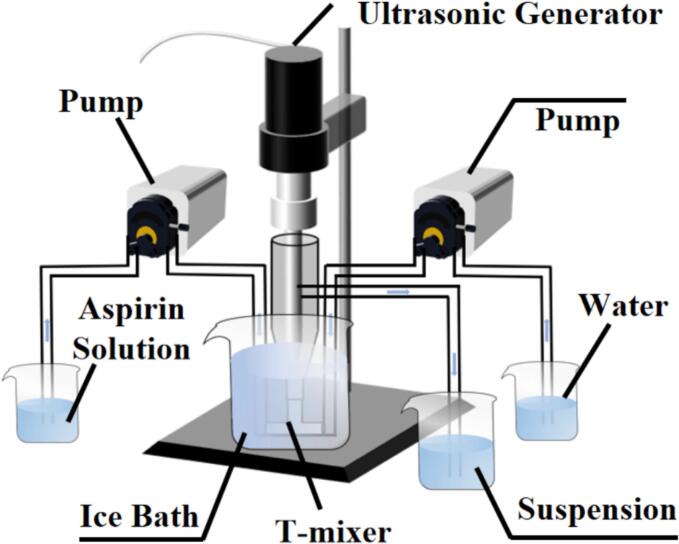
Experimental setup for ultrasound intensified anti-solvent crystallization (UIAC).

Aspirin is soluble in ethanol and slightly soluble in water. As water and ethanol are miscible, ethanol and water are chosen as the positive solvent and anti-solvent for the experiments to prepare aspirin suspension. Certain amount of aspirin was added and fully dissolved in ethanol to prepare aspirin ethanol solution. Fig. 1 shows the ultrasound-intensified turbulence microreactor [33,34] we developed for UIAC, including two peristaltic pumps (BT100FJ, Baoding Chuangrui, China), a T-mixer, a continuous kettle reactor, and an ultrasonic generating device (Better-1200ST, Fangxu Technology Shanghai, China). One side of the T-mixer tube is connected to the inlet end of the continuous kettle reactor, which is placed in ice bath (10 °C) to achieve crystallization in supercooling condition. The liquid volume is maintained at 40 mL. The ultrasound irradition temperature ranged from 41.4 to 70.6 s depending on the feeding rate of solvent. Meanwhile, the other end is connected to two peristaltic pumps through a silicone tube. The other side of the two peristaltic pumps is aspirin ethanol solution and pure water. The joints of the three-way tube and the kettle reactor are immersed in the ice water bath. The cylindrical probe of the ultrasound generator connected with the interior of the kettle reactor. The inner diameter of the silicone tube is 2 mm. The speed of the peristaltic pump is set at a certain value (100 rmp = 28 mL/min). When the solution is filled with the kettle reactor and the liquid flows out at the outlet, and the suspension is collected when the white crystal particles flow out. The preparation conditions for all samples are shown in Table S1. Then aspirin powder could be obtained after drying. To reduce electrostatic effects and improve powder fluidity, the integration of lactose monohydrate (L150) and L-leucine (Leu) as supplementary materials was necessary. Aspirin inhalation formulation was prepared by mixing aspirin powder with L150 and Leu. The mixing ratio was set as in Table S2.

#### 2.2.2 Evaluation the significance of process parameter via machine learning

To assess the influence of various factors such as concentration, ultrasonic power, ratio between water and ethanol, rate of water, and spray pressure on particle size, we employed a machine learning methodology, specifically leveraging a decision tree regressor, which is shown in our previous studies [35,36]. These factors were considered independent variables or features, while particle size metrics served as the dependent variable or target. To construct a robust model and mitigate overfitting, we set the minimum depth of the decision tree to 4. Additionally, other hyperparameters of the decision tree were fine-tuned to minimize mean square error and optimize model performance.

#### 2.2.3 Evaluation of calorimetric power

Pure water (40 mL) was used to evaluate the thermal energy received by the medium at different ultrasound power to determine the calorimetric power (Tables S3 and S4). The water in the conical flask is irradiated by ultrasonic wave piezoelectric vibrator with different power (300 W, 600 W, 900 W, 1200 W and 1500 W). The value of temperature is recorded every 1 min by thermometer. The experiment was repeated for three times. The power of ultrasound dissipated inside the conical flask is calculated by the following equation [35]:

(2.1) $$Powerofultrasound\left(P\right)=mC_{P}\left(\mathrm{dT}/dt\right)$$

where *m* is the mass of water (0.04 kg), *C_P_* is the specific heat capacity of water (4.2 kJ/kg·K), *dT/dt* is the temperature change per second.

### 2.3 Physicochemical characterization

#### 2.3.1 Particle size distribution

The particle size and distribution of inhalable powder were measured by HELOS-RODOS/L laser diffraction (New Partek, Inc) under 2.0 bar in triplicate (n = 3). The value of the span was calculated employing the following Eq. (2.2):

(2.2) $$\mathrm{Span}=\frac{\left(D_{90}-D_{10}\right)}{D_{50}}$$

#### 2.3.2 Morphology assessment

Field Emission Scanning Electron Microscope (SEM, Zeiss Gemini 300, Germany) was used to investigate the morphology of the samples. The samples were dispersed on conductive adhesive and imaged in the sample compartment at 1.0 kV.

#### 2.3.3 Density determination

As one of the most basic and important physical properties, the density of powder directly affects aerodynamic performance and delivery efficiency significantly. The loose density (bulk density, *ρ_B_*) and vibration density (tap density, *ρ_T_*) were determined by measuring cylinder method in this study. First, certain amount of dry powder sample was weighed and gently filled in a 5 mL measuring cylinder. Accurately recorded the current loose volume (bulk volume, *V_B_*). Then, gently tap the measuring cylinder until the volume of the dry powder sample does not change, and the vibration volume (tap volume, *V_T_*) is recorded. Finally, the sample's loose density and vibration density are calculated according to Eqs. (2.3) and (2.4). The measurement was repeated three times for each prescription.

(2.3) $$\rho_{B}=\frac{m}{V_{B}}$$

(2.4) $$\rho_{T}=\frac{m}{V_{T}}$$

#### 2.3.4 Flowability measurement

To evaluate the powder flowability, Karl compression index (CI) and the Housner ratio (HR) were calculated by the following Eqs. (2.5) and (2.6):

(2.5) $$\mathrm{CI}=\frac{\left(\rho_{T}-\rho_{B}\right)}{\rho_{T}}\times 100\%$$

(2.6) $$\mathrm{HR}=\frac{\rho_{T}}{\rho_{B}}$$

#### 2.3.5 Differential scanning calorimetry (DSC)

To evaluate the thermal response profile, inhalable powder (2 to 3 mg) was transferred into a 40 μL aluminum pot, then tested by differential scanning calorimetry (DSC, Mettler Toledo, Milan, Italy) under N_2_ atmosphere (10 °C/min, from 25 to 260 °C).

#### 2.3.6 Powder X-ray diffraction (PXRD)

The polymorphism of as-prepared particles was evaluated by PXRD (Bruker D8, Karlsruche, Germany) by using Cu Kα target ray scanning. The tube current and pressure were 40 mA and 40 KV.

#### 2.3.7 Fourier transform infrared analysis (FTIR)

In this study, Thermo Fisher's Nicolet iS5 FTIR spectrometer was used to investigate the chemical structure of samples. The specific operation method is as follows: place the dry powder particles to be tested on the ATR template detection stage of the infrared spectrometer, press down the sample fixation probe to lock, and set the number of scans of the instrument to 32, then start the test.

### 2.4 Aerodynamic performance measurement

The aerodynamic performance was evaluated with NGI (Logan, USA). The particles were loaded at a dose of 10 ± 0.5 mg/grain intogelatin capsule. Ten capsules were used in each experiment, and the air flow rate was maintained at 60 L/min for 4 s. The NGI plate was firstly coated with Tween 20 in ethanol (10 %, v/v) for bounce or re-entrainment prevention. The powders collected by each NGI stages was rinsing with ethanol and determined by HPLC. The percentage of loaded powder in the capsule was detemined by emitted dose (ED). The fine particle fraction (FPF) was calculated as the percentage of the powder emitted from the inhaler with an aerodynamic diameter less than 5 μm. The mass median aerodynamic diameter (MMAD) and the geometric standard deviation (GSD) were calculated by Copley Inhaler Testing Data Analysis Software Version 3.10. Each formulation was evaluated in triplicate [37].

### 2.5 In-vitro release kinetics

In terms of in-vitro release kinetics study, 10 mg of powders was dispersed into 30 mL phosphate buffer (PBS), artificial lung fluid (ALF) and Gamble’s solution (GMB) (Table S5) [38]. The dispersion was incubated in a horizontal-shaking water bath at 37 °C. At scheduled time intervals were set in 3, 6, 9, 12, 15, 20, 30, 45, 60, 90, 120, and 180 min. 0.5 mL of medium was taken out and analyzed by HPLC. Meanwhile, 0.5 mL of fresh-release medium was added into maintain the total solution volume.

### 2.6 Stability test

The stability of the inhalable powder samples A3, F3, and F6 was performed under two conditions (25 °C and 60 % RH; 40 °C and 75 % RH) for one month. Then the samples were characterized by NGI, XRD and DSC.

### 2.7 Cytotoxicity

The cytotoxicity of the particles on cell level was evaluated by human alveolar basal epithelial A549 cell line via MTT test. Before using in viability assays, cells were grown at 37 °C in 5 % CO_2_ atmosphere for 12 h. The samples were dissolved in medium to prepare drug solutions with different concentration. Then each well was added 100 μL drug solution. After incubating for 24 h, each well was treated with 10 µL of MTT solution (5 mg/mL) and continued incubation. After 4  h, the medium was removed, while the crystals generated were solved with 150 μL DMSO. The relative cell viability (%) was calculated as the following Eq.n (2.6):

(2.6) $$\mathrm{Cell}\;viability\;\left(\%\right)=\frac{\mathrm{Value}\;of\;treatment\;group}{\mathrm{Value}\;of\;control}\times 100\%$$

### 2.8 Drug permeability

The drug permeability and mechanism of drug dissolution, deposition, and diffusion/transport in the lung was investigated by in-vitro aerosol testing apparatus with Calu-3 cell line. The NGI plate was modified to allow the attachment of transwell containing the Calu-3 epithelial cell line. The particles deposited on outer surface of the transwell were washed away, then transferred to 12-well plate containing freshly preheated HBSS (600 μL). The initial amount of particles deposited on the cell layer could be calculated based on the total amount of drug that passed through the cell monolayer, retained on the monolayer and inside the cells.

### 2.9 Pharmacokinetic study

Twelve SD rats (male, 180–200 g body weight) were randomly assigned to 2 groups (n = 6), including the oral (gavage) and inhale at 9 mg/kg groups, equivalent to a human equivalent dose of 100 mg and an average body weight of 60 kg. The blood samples (500 μL) were immediately drawn and collected into EDTA-contained tubes after intratracheal administration. To extract salicylic acid (SA) from plasma, 200 μL of serum from plasma samples was mixed with 400 μL of acetonitrile, vortex shaking for 30 s, and centrifugation at 10,000 rpm for 10 min. The supernatant was filtrated with 0.22 µm filter head for HPLC analysis. The concentration of SA in plasma was determined by Agilent 1260 Infinity II LC System and Agilent C8 column (5 μm, 250 mm × 4.6 mm, Agilent Technologies Inc., USA). The mobile phase consisted of aqueous acetic acid solution (0.25 %) and acetonitrile (65:35, v/v). The flow rate of mobile phase was kept at 1.0 mL/min. The detection wavelength and column temperature was maintained at 303 nm and 25 °C, respectively.

### 2.10 Antiplatelet aggregation

The experimental grouping is the same as in section 2.9. According to the results of pharmacokinetic experiments in section 3.8, the T_max_ of inhalation group was 10 min. Therefore, The blood samples (2 mL) were immediately drawn and collected into 3.8 % sodium citrate anticoagulant tubes at 10 mins, 1 h and 2 h after the administration of the drug, placed at room temperature for platelet aggregation assay, and the assay was completed within 2 h after blood collection.Arachidonic acid (AA) and adenosine diphosphate (ADP)-induced platelet aggregation was measured by conventional light transmission aggregometry (LTA), and the final result was recorded as the maximum platelet aggregation rate. Platelet-rich plasma (PRP) and platelet-poor plasma (PPP) were obtained by centrifugation. The platelet aggregometer (Beijing Mindray, lby-nj4, China) was calibrated using the PPP, and platelet aggregation rates were assessed by introducing PRP of different groups together with aggregation inducers, arachidonic acid (AA) or adenosine diphosphate (ADP). Aggregation was measured by conventional light transmission aggregometry (LTA), and the final results recorded as the maximum platelet aggregation rate.

### 2.11 In vivo safety

Mice were randomly divided into control, oral and inhale groups (n = 6 per group). In the oral and inhale groups, mice were administrated with aspirin (9 mg/kg/day) for 7 days. In control group, mice were non-treated. After 7 days, the mice were euthanized. Their main organs were isolated and stained with hematoxylin and eosin (H&E).

## 3 Results and discussion

### 3.1 Investigation of preparation condition

The particle size distribution of excipient-free aspirin powders are shown in Table S1. The effects of ultrasonic power, drug concentration, antisolvent-to-solvent ratio, and anti-solvent rate of the UIAC process, were systematically investigated.

#### 3.1.1 Effect of ultrasound intensity

**Fig. 2:**
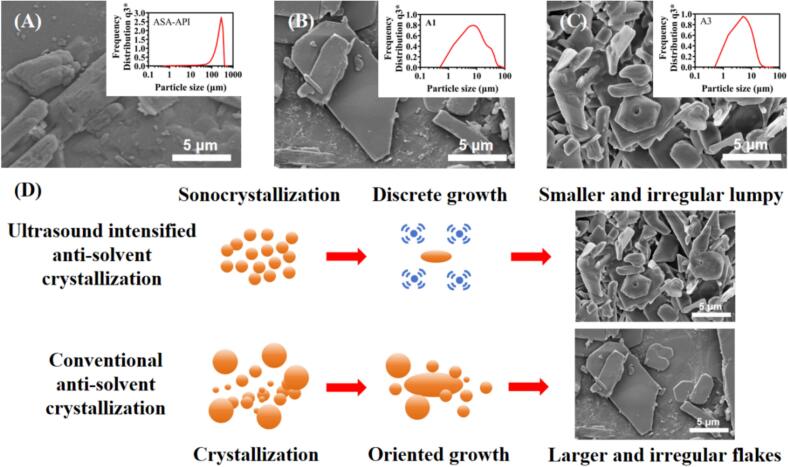
Morphology and particle size distribution of (A) aspirin API; (B) sample A1; (C) sample A3; (D) mechamism of ultrasound intensified anti-solvent crystallization and conventional anti-solvent crystallization on the formation of aspirin powder.

**Fig. 3:**
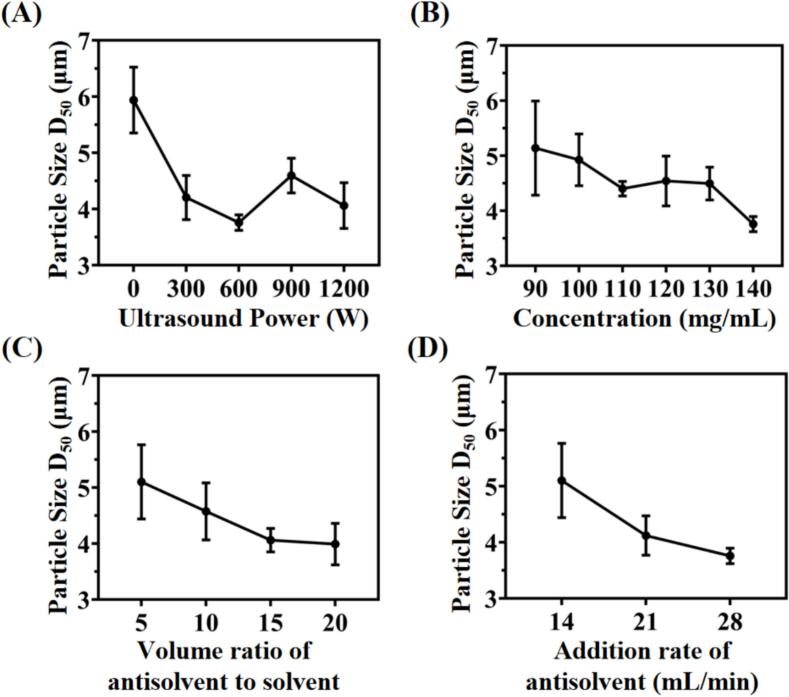
Relationship between particle size with (A) ultrasonic power; (B) aspirin concentration; (C) volume ratio of antisolvent to solvent; (D) addition rate of antisolvent.

The effect of ultrasound intensity on particle size was systematically investigated at different ultrasonic power ranging from 0 W to 1200 W, while maintaining the drug concentration (140 mg/mL), volume ratio of antisolvent-to-solvent (v_anti_/v_sol_ = 10), and addition rate of antisolvent (28 mL/min) at same level. As shown in Fig. 2, the aspirin API particles (ASA-API) showed rod-shape with rough surface, and particle size ranged from 80 to 300 μm (Fig. 2A). Meanwhile, the sample A1 obtained in the absence of ultrasound was an irregular flake with broader particle size distribution ranging from 1.48 ± 0.13 to 22.31 ± 2.98 μm (Fig. 2B). In contrast, Fig. 2C showed that the sample A3 obtained with 600 W ultrasonic power had an irregular lumpy morphology with narrower particle size distribution (1.19 ± 0.04 to 9.55 ± 0.80 μm) compared to A1. Thus, the introduction of ultrasonic radiation not only achieved uniform particle size distribution, but also affected the morphology of the particles. Moreover, in Fig. 3A, increasing ultrasound intensity from 0 W to 600 W leads to a reduction of particle size (D_50_) from 5.94 ± 0.95 μm (A1) to 3.82 ± 0.03 μm (A3). After that, further improving ultrasonic intensity to 1200 W resulted in the value of D_50_ slightly raising to 4.06 ± 0.59 μm (A5). This phenomenon could be attributed to that, ultrasonic radiation could enhance micro-mixing between positive and negative solvents, and rapidly generate high degree of supersaturation and nucleation rate. Meanwhile, cavitation bubbles could be formed by ultrasonic radiation, resulting in the reduction of nucleation energy barriers and bonding force. Therefore, the generation of agglomerates were inhibited among as-synthesized aspirin crystals. Besides, shock wave could be generated by the collision between positive and negative solvents, while the turbulence could be generated by ultrasonic cavitation. The synergistic effect of shock wave and turbulence significantly reduced the thickness of diffusion layer around the crystal surface. The high-speed collisions between crystal molecules increased the number of crystals and inhibited crystal agglomeration. Thus, the shape and size of as-synthesized particles were affected, and facilitated the generation of smaller solid particles with narrower particle size distribution [39,40] (Fig. 3A). Further increasing of ultrasonic irradiation power (600–1200 W) facilitated the breakage of crystals, and generated a reaction environment with high temperature even in ice-water bath. The temperature of pure water could increase 32.3–72.7 °C in 4 mins when applying ultrasound power (300–1500 W). Although the reaction equipment was immersed in ice bath (around 10 °C), the transient high temperature induced by ultrasound still triggered partial dissolution and bonding among particles. Therefore, the re-dissolution of particles was enhanced in this situation [41], and the optimized ultrasonic power in this experiment was 600 W.

#### 3.1.2 Effect of aspirin concentration

Fig. 3B showed the relationship between aspirin concentration and particle size. The experiments were carried out at different drug concentrations (90, 100, 110, 120, 130, 140 mg/mL) while maintaining the volume ratio of antisolvent-to-solvent (v/v = 10), the addition rate of antisolvent (28 mL/min), and ultrasonic power (600 W). When increasing aspirin concentration from 90 mg/mL to 140 mg/mL, the value of D_50_ kept decreasing tendency and reduced from 5.14 ± 0.85 μm (A10) to 3.82 ± 0.03 μm (A3). This phenomenon was due to the high supersaturation resulting from mixing of aspirin solution with high concentration, so that higher driving force can be achieved and lead to rapid nucleation. The large amount of nuclei could be generated in an extremely short time, and facilitated the formation of fine aspirin particles [42]. Otherwise, aspirin solution with lower concentration led to the production of large crystals due to low degree of supersaturation [43].

#### 3.1.3 Effect of antisolvent-to-solvent ratio

The effect of the antisolvent-to-solvent ratio was evaluated with different antisolvent-to-solvent ratios (v/v = 5, 10, 15, 20) while maintaining the drug concentration (130 mg/mL), ultrasonic power (600 W), and the addition rate of antisolvent (28 mL/min). As shown in Table 1 and Fig. 3C, the particle size (D_50_) reduced from 6.91 ± 2.19 μm (A17) to 4.42 ± 0.96 μm (A12) when the antisolvent-to-solvent ratio improved from 5 to 20. The reason can be attributed to the higher supersaturation and nucleation rate caused by the increasing of antisolvent-to-solvent ratio, so that interface could be formed rapidly between solvent and anti-solvent. Subsequently, the particle growth was inhibited, resulting in the presence of a large amount of smaller nuclei [44]. Conversely, decreasing the anti-solvent ratio could increase particle size due to the slower crystallization rate [45].

**Table 1:** Aerosol performance characteristics of sample A3, F3, and F6. (mean ± SD, n = 3).

Sample	ASA (W/W %)	Leu (W/W %)	L150 (W/W %)	ED (%)	FPF (%)	MMAD (µm)	GSD
A3	100	0	0	86.6 ± 7.6	10.4 ± 0.9	5.8 ± 0.5	1.8 ± 0.1
F3	35	5	60	97.5 ± 2.2	45.9 ± 0.6	4.6 ± 0.1	1.6 ± 0.1
F6	65	5	30	95.4 ± 1.6	38.9 ± 1.7	4.9 ± 0.1	1.6 ± 0.1

#### 3.1.4 Effect of the addition rate of antisolvent on particle size

Fig. 3D revealed how the addition rate of anti-solvent influenced particle size while maintaining the drug concentration (130 mg/mL), ultrasonic power (600 W), and volume ratio of antisolvent-to-solvent (10:1). As the addition rate of anti-solvent increased from 14 to 28 mL/min, the particle size D_50_ decreased from 6.25 ± 0.95 μm (A24) to 4.49 ± 0.30 μm (A6). In UIAC, the counter-solvent water was mixed with the aspirin ethanol solution in the T-type connector (shown in Fig. 1). The joint actions of the high impinging stream rate and ultrasound irradiation can induce turbulence through the impinge streams, then enhance internal micro-mixing and reduce mass transfer resistance. Meanwhile, the rapid generation of high supersaturation induces a higher nucleation rate. Besides, higher flow rate mixing enhanced shear force, and contributed to the formation of particles with smaller size and narrower distribution. At lower flow rate, less nucleation sites formed due to lower mixing efficiency between solvent and antisolvent, resulting in the formation of larger crystals [44]. Meanwhile, the continuous unimpeded operation is ensured in the high flow rate state, and the low flow rate state, the inhomogeneity of the particle growth results in larger particles that may clog the T-pipe [41].

#### 3.1.5 Evaluation of key process parameter via machine learning

**Fig. 4:**
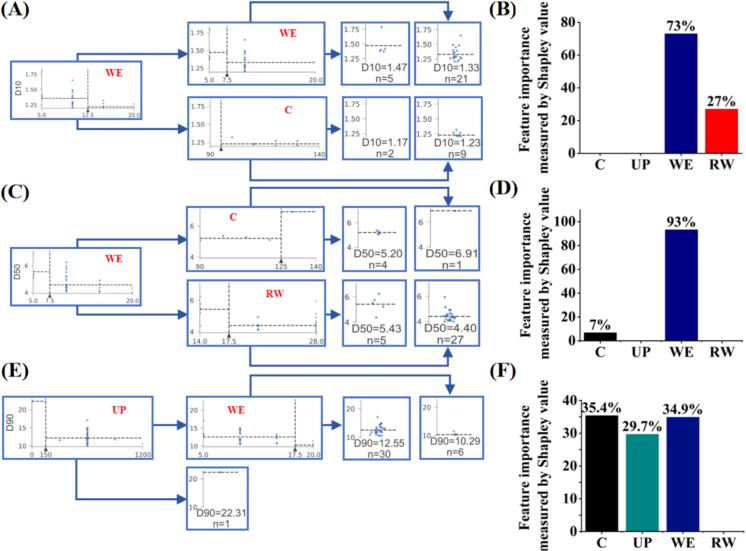
Result of mechine learning for screening key process parameters. Schematic diagram of the decision tree model and the importance of process parameters based on Shapley values: (A-B) D_10_; (C-D) D_50_ and (E-F) D_90_. (C = Concentration; UP = Ultrasonic Power; WE = Volume Ratio of Water to Ethanol; RW = Addition Rate of Water).

The preparation conditions of 37 aspirin powders ranged from 1 to 5 μm were shown in Table S1. A machine learning method was applied to screen the key process parameters for each process parameter involved in the production process. Fig. 4 represented the effect of process parameters on particle size D_10_, D_50_, and D_90_. In terms of the D_90_ value of aspirin powders (Fig. 4E and F), the concentration of aspirin (C) had the greatest effect (35.4 %), followed by the ratio of antisolvent to solvent (WE, 34.9 %) and ultrasonic power (UP, 30 %). In contrast, it is obvious to see that the ratio of antisolvent to solvent (WE) had the greatest effect on D_10_ and D_50_ value, which is 73 % and 93 %, respectively. Thus, the particle size distribution of aspirin inhalation powder was most affected by the volume ratio of anti-solvent to solvent.

### 3.2 Physicochemical characterization of aspirin inhalation formulation

#### 3.2.1 Flowability

In Table S6,15 inhalable powders were selected for the measurement of flowability. However, all excipient-free aspirin powder prepared in this work showed greater electrostatic effect and poor flowability, resulting in higher value of CI (>50 %) and HR (>2). Sufficient flowability is essential for drug transportation to the lower respiratory tract. The flowability of the as-synthesized powder could be improved significantly via the addition of excipients, which reduced the interaction force between particles [46]. Hence, the as-prepared aspirin powder was mixed with excipients such as lactose monohydrate (L150) and L-leucine (Leu) to reduce electrostatic effect and improve pulmonary delivery efficiency.

As shown in Table S7, the uniformity of drug content was within the acceptable range of 85–115 % (British Pharmacopoeia Volume V Appendix Xll Content Uniformity), thus all the blended formulations could be regarded as homogeneous formulation mixtures. Moreover, the CI index value decreased from 62.03 ± 4.30 % (A3) to 27.24 ± 0.64 % (F3) and 27.50 ± 1.48 % (F6), the HR value decreased from 2.63 ± 0.30 (A3) to 1.37 ± 0.01 (F3) and 1.38 ± 0.03 (F6). In the aspirin inhalation formulation, larger L150 particles could be regarded as carriers, while Leu and ASA-DPIs tended to be attached on the surface of L150 particles. Surface coverage provided a physical barrier that increased the separation distance between adjacent micronized particles, resulting in the reduction of inter/intra particulate interactions [47]. Due to the smaller particle size, Leu filled in the irregular pores of L150 carriers as active site occupation, and optimized the dispersion performance [48]. Moreover, Leu acts as a dispersion enhancer with unique amphiphilic properties that make its hydrophobic domain tend towards aspirin particles, while its hydrophilic domain tends towards lactose particles, thereby modifying the forces of interaction between the particles and improving the fluidity of the powder. Hence, the aspirin powder could adhere tightly to the carrier during transportation, and de-agglomerated from the carrier to enter the bronchial branches easily during pulmonary delivery.

#### 3.2.2 Morphology and particle size distribution

**Fig. 5:**
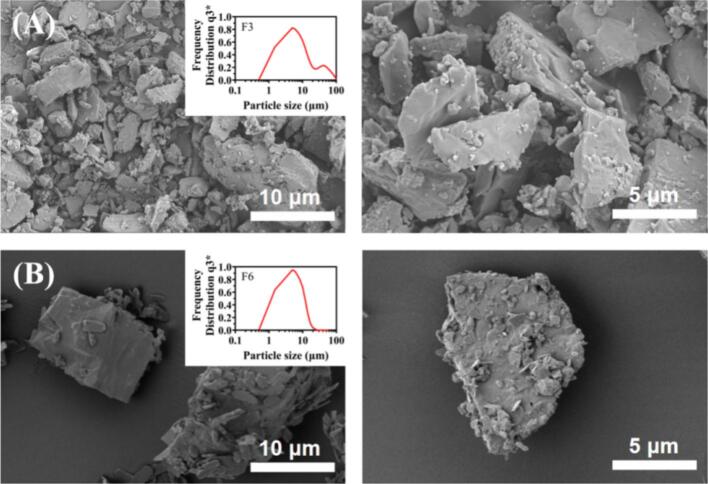
Morphology and particle size distribution of (A) Sample F3; (B) Sample F6.

Fig. 5 showed the particle size distribution and morphology of the inhalation formulation F3 and F6. It was clearly visible that the physical mixtures (F3 and F6) were formed by mixing excipient-free aspirin particles (A3) with Leu and L150. where aspirin particles and Leu (1–3 μm) adhered on the surface of irregularly shaped carrier L150 (10–50 μm), filling the irregular areas, reducing contact between aspirin particles, and weakening cohesion. Due to the larger amount of L150 (60 %), smaller aspirin and Leu particles were not enough to cover the L150 surface, so that the particle size distribution of sample F3 revealed bimodal pattern (Fig. 5A). In contrast, the PSD result and SEM images of sample F6 (Fig. 5B) showed more small aspirin and Leu particles adhering to the surface of large L150 particles, indicating that less L150 (30 %) is sufficient to provide attachment points for the small particles.

#### 3.2.3 Differential scanning calorimetry (DSC)

**Fig. 6:**
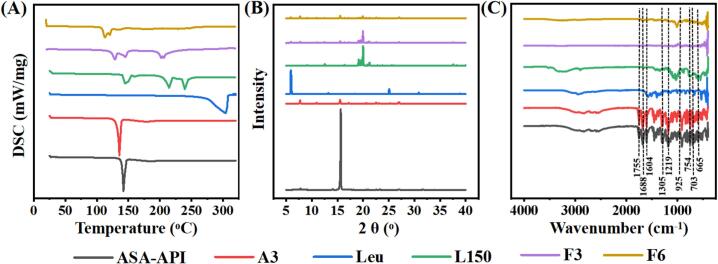
(A) DSC; (B) XRD; (C) FTIR results of aspirin API, excipient-free aspirin DPIs (A3), lactose monohydrate (L150), L-leucine (Leu), and aspirin inhalable powder (F3 and F6).

To investigate the thermal stability, DSC analysis is performed by continuously measuring changes in temperature and energy over time in solid phase transition [49]. Fig. 6A showed the melting point of aspirin API, aspirin excipient-free DPIs (A3), L-leucine (Leu), lactose monohydrate (L150), as well as the DPIs sample F3 and F6. The melting peak of excipient-free aspirin powder (A3) was 135.6 °C, slightly lower than the melting peak of ASA-API (142.1 °C). The shift of endothermic peak to lower temperature indicated the reduction of crystallinity. The heat absorption peaks appeared at 128.7 °C, 144.8 °C and 205.8 °C in sample F3. Similar to L150, the shift of peak might be caused by the physical interactions or lack of resolution. The sample F6 showed a broad thermal absorption peak only at 112.7 °C, probably due to the formation of hydrogen bond among the drug, L150 and Leu.

#### 3.2.4 X-ray diffraction analysis (XRD)

The XRD patterns of aspirin API, aspirin excipient-free powder (A3), L-leucine (Leu), lactose monohydrate (L150), as well as the sample F3 and F6 are shown in Fig. 6B. The XRD pattern of ASA-API showed some sharp peaks, especially at 2θ≈15°, which confirmed the crystalline structure of ASA-API. In contrast, the XRD pattern of aspirin excipient-free powder (A3) exhibited the same pattern with lower intensity. This is mainly due to the decrease in particle size, and also indicates a decrease in crystallinity of the micro-aspirin [50]. The comparison between XRD patterns for ASA-API, DPIs sample A3, F3 and F6, had minor diffractions at 2θ≈8.1°, 20.98°, 22.86°, 23.02°, and 26.82°, which is the main structural characteristic of aspirin. Furthermore, the major peak from aspirin can be observed at 15° in sample F3 and F6, suggesting that the addition of Leu and L150 did not affect the crystallinity of the aspirin.

#### 3.2.5 Fourier transform infrared spectrum (FTIR)

The infrared spectrum of samples (ASA-API, sample A3, F3 and F6) and excipient (Leu and L150) were measured by FTIR. Generally, aspirin has three main specific functional groups. The spectrum displayed bands at 1735–1750 cm^−1^ due to the ester group (R-C= O-O-R) stretching, and bands ranging from 2500 to 2600 to 3100–3200 cm^−1^ due to the carboxylic acid group (COOH) stretching. Besides, the central benzene ring produced carbonyl C=O group stretches (about 1710–1780 cm^−1^), a medium peak for C=C (about 1500––1700 cm^−1^) and sharp C-H stretching peaks (∼3030–3040 cm^−1^) [51]. As shown in Fig. 6C, the principle peaks of aspirin, which are at 1183, 1688, 1604, 1305, 1755, 1219, 925, 754, 703 and 665 cm^−1^ were present in DPIs samples A3, F3 and F6. Meanwhile, no specific chemical changes or interactions were noticed among the drug and excipients from the developed DPI formulations. Therefore, the excipients used in inhalable powder were compatible with active drug.

### 3.3 In vitro aerosol performance

**Fig. 7:**
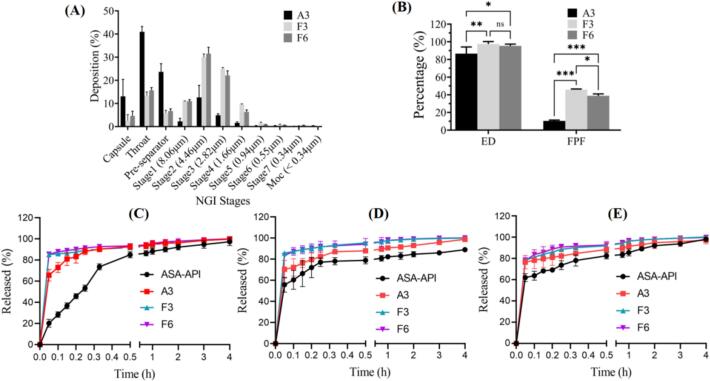
(A) Distribution of particle deposition across components of the NGI assembly for DPIs sample A3, F3, and F6; (B) ED and FPF for DPIs sample A3, F3, and F6. (Mean ± SD, n = 3); (C–E) Dissolution profiles of ASA-API, DPIs A3, F3 and F6 in PBS (C), GMB (D) and ALF (E).

The aerosol performance of DPIs sample A3, F3, and F6 were evaluated through NGI, and shown in Table 1 and Fig. 7. The ED value of F3 (97.54 ± 2.22 %) and F6 (95.38 ± 1.65 %) was higher than A3 (86.58 ± 7.57 %), which corresponded to fairly lower dose loss when the F3 and F6 were inhaled. The FPF revealed the ability of particles to reach the respirable region with an aerodynamic size about ≤5.0 μm The FPF of F3 (45.86 ± 0.63 %) and F6 (38.91 ± 1.74 %) was significantly higher than that of A3 (10.40 ± 0.85 %). While the MMAD of F3 (4.58 ± 0.03 μm) and F6 (4.94 ± 0.13 μm) was smaller than A3 (5.76 ± 0.52 μm), which were in the respirable range (1 to 5 µm) and correlated with FPF. The GSD of F3 (1.63 ± 0.01) and F6 (1.64 ± 0.03) was lower than A3 (1.82 ± 0.13), indicating reduced polydispersity of the particle size distribution.

The deposition results for each collection tray were shown in Fig. 7A. The deposition in the capsule of F3 and F6 were less than 5 %, whereas the A3 had 13.05 ± 7.37 %, indicating that F3 and F6 were easily atomized by the device. Sample A3 was mainly deposited in the throat (40.94 ± 2.32 %) and pre-separator (23.67 ± 3.51 %), which may be related to the greater electrostatic effect of the powder. In contrast, the mass of F3 and F6 collected from stage 2 to stage 7 was higher than that of A3, and mainly deposited in stage 2 and stage 3. The FPF of F3 and F6 can reach up to 40 % while A3 was only around 10 %, indicating the addition of L150 and Leu helped to reduce electrostatic force and improve the aerosol performance.

### 3.4 In vitro dissolution rate

Fig. 7C–E showed the dissolution profiles of ASA-API, sample A3, F3 and F6 in PBS, and simulated lung fluids (GMB and ALF). As shown in Fig. 7C, samples A3, F3 and F6 dissolved almost completely, while only 85 % of ASA-API dissolved within 1 h in PBS. In GMB solution (Fig. 7D), samples F3 and F6 dissolved almost completely within 1 h, but only 80 % of ASA-API and 90 % of sample A3 could be dissolved. Besides, 95 % of sample F3 and F6 dissolved in ALF within 1 h, which was higher than that of ASA-API (85 %) and sample A3 (90 %). After 4 h, samples A3, F3 and F6 offered better drug dissolution profile than ASA-API, and dissolved completely in PBS, GMB and ALF. Based on Noyes-Whitney equation (Eq. (3.1)), the improved of dissolution of F3 and F6 in PBS, GMB and ALF can be attributed to reduced aspirin particle size, increased surface area, and enhanced interaction between smaller particles and solvent. Besides, the presence of hydrophilic L150 and amphiphilic Leu reduced the cohesion between particles, thus improved the solubility of aspirin [52].

(3.1) $$\frac{\mathrm{dC}}{\mathrm{dt}}=K_{D}A\left(C_{s}-C_{t}\right)$$

### 3.5 Stability of aspirin DPIs

**Fig. 8:**
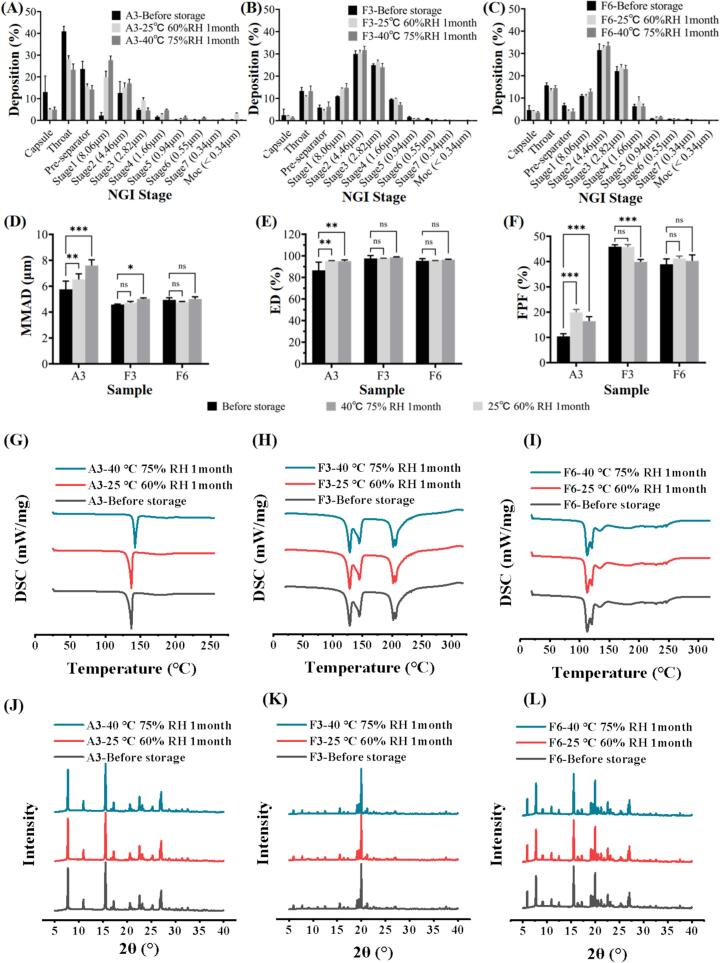
The stability of sample A3, F3 and F6 performed at 25 °C, 60 % RH and 40 °C, 75 % RH for 1 month: (A–C) NGI deposition; (D-F) MMAD, ED and FPF before and after storage; (G-I) DSC and (J-L) XRD patterns.

To evaluate the stability of DPIs, samples A3, F3 and F6 were stored at 25 °C, 60 % RH, and 40 °C, 75 % RH for one month. As shown in Fig. 8A. the amount of A3 in the capsule, throat, and pre-separator was greatly reduced after storage, and slightly increased from stage 2 to stage 7, which may be related to the decrease in electrostatic of the powder due to higher humidity. Fig. 8B-C show that the amounts of F3 and F6 had no significant changes in each collection tray after one month. Fig. 8D-F showed that F3 had no significant changes in terms of MMAD, ED and FPF 25 °C, 60 % RH. However, after storing at 40 °C, 75 % RH, the F3 powder might be agglomerated under high temperature and humidity, resulting in the increasing of MMAD and reduction of FPF. In contrast, MMAD, ED, and FPF of sample F6 had no significant changes after storage. The DPIs samples were further measured by XRD and DSC to evaluate the stability. Fig. 8G-I showed that there was no significant change in the melting peaks of A3, F3, and F6 before and after storing at 25 °C, 60 % RH, and 40 °C, 75 % RH for one month. The XRD result of sample A3, F3, and F6 exhibited no significant changes (Fig. 8J-L), indicating there was no change in crystalline state during the storage process, which was also confirmed by DSC (Fig. 8G-I). The above results indicate the better stability of F6 under the two storage conditions tested, no chemical degradation of F6 and phase transition was observed within 1 month. The discussion above demonstrated better in vitro aerosol performance and dissolution behavior of samples F6 and F3. Furthermore, sample F6 revealed improved stability after storing at 25 °C, 60 % RH and 40 °C, 75 % RH for one month. It is expected that sample F6 could be delivered effectively to the peripheral lung and dissolved rapidly. Therefore, sample F6 was selected for further in-vitro and in-vivo biological evaluation. The discussion above demonstrated better in vitro aerosol performance and dissolution behavior of samples F6 and F3. Furthermore, sample F6 revealed improved stability after storing at 25 °C, 60 % RH and 40 °C, 75 % RH for one month. It is expected that sample F6 could be delivered effectively to the peripheral lung and dissolved rapidly. Therefore, sample F6 was selected for further in-vitro and in-vivo biological evaluation.

### 3.6 In vitro cell viability and drug permeability through cell

**Fig. 9:**
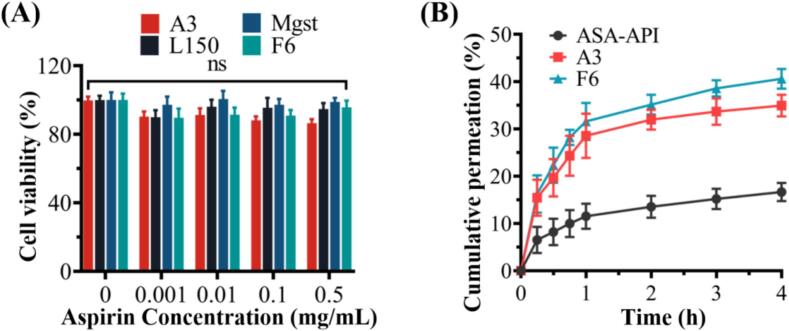
(A) Viability of A549 cells after 24 h exposure to sample A3, Leu, L150 and F6 (mean ± SD, n = 6); (B) cumulative percentage of drug permeated through Calu-3 cell monolayer as a function of time for ASA-API, sample A3 and F6 (mean ± SD, n = 3).

The in vitro cell viability was evaluated by A549 human alveolar epithelium cell line and determined by MTT assay, which is commonly used in inhalation toxicology [52]. The A549 cell viability values of aspirin excipient-free DPIs (A3), L150, Leu, and sample F6 were all higher than 90 % (Fig. 9A), indicating that sample F6 is safe and feasible for pulmonary inhalation administration.

The permeability were investigated via air-interface Calu-3 epithelial cell line and TEER to evaluate the potential of the inhalable powder in barrier disruption of the alveolar epithelium. Before the measurement, the TEER values of all the chambers were in the range of 300–1000 Ω/cm^2^ (Table 2), After 4 h treatment, the TEER value of all the chambers reduced slightly (<20 Ω/cm^2^), indicating the powder sample was noncytotoxic on the integrity of the alveolar epithelial cell layer. Fig. 9B showed the drug permeability (apical to basal) through the air-interface Calu-3 epithelial cell line. It showed an initial ‘burst’ release followed by a linear profile with time up to 1 h and then plateau gradually. After 4 h, the cumulative permeation of A3 (69.87 ± 4.60 %) and F6 (77.15 ± 2.79 %) was higher than that of aspirin API (35.27 ± 4.31 %), indicating F6 had the best permeability through the air-interface Calu-3 epithelial cell line. As shown in Table 3, the amount of aspirin API remained on the cell surface (38.95 ± 3.12 %) was higher than A3 (11.50 ± 1.11 %) and F6 (10.73 ± 1.96 %). Meanwhile, The amount of intracellular ASA-API (25.78 ± 0.45 %) was higher than for A3 (18.63 ± 0.55 %) and F6 (12.12 ± 0.84 %). These results demonstrated the good permeability of aspirin in Calu-3 cell monolayer, as well as the greater cellular permeability of F6, which is correlated with in vitro aerosol performance and dissolution rate.

**Table 2:** Trans-Epithelial Electrical Resistance (TEER) (Ω/cm^2^) of Calu-3 cells and aspirin distribution at 4 h after treatment by ASA-API, sample A3 and F6.

Sample	TEER before deposition (Ω/cm^2^)	TEER 4 h after deposition (Ω/cm^2^)	4 h cumulative drug permeated (%)	Cell surface (%)	Intracellular (%)
ASA-API	427 ± 15	407 ± 10	35.27 ± 4.31	38.95 ± 3.12	25.78 ± 0.45
A3	405 ± 12	403 ± 5	69.87 ± 4.60	11.50 ± 1.11	18.63 ± 0.55
F6	392 ± 7	379 ± 6	77.15 ± 2.79	10.73 ± 1.96	12.12 ± 0.84

**Table 3:** Estimated SA serum PK parameters applying NCA sparse data analysis using Phoenix® for inhaled and oral aspirin sample F6.

Parameter	C_max_ (µg/mL)	T_max_ (h)	T_1/2_ (h)	AUC_0-∞_ (µg·h/mL)	Relative bioavailability (%)
Oral	26.21 ± 4.90	0.5	4.12	121.36	−
Inh	46.05 ± 2.60	0.17	2.69	231.88	1.91

### 3.7 In-vivo pharmacokinetics study

To evaluate the advantages of aspirin inhalable powders, the in-vivo pharmacokinetic study on oral and inhalation administration was performed in rats (Fig. S1A-B). The dose of F6 is 13.85 mg/kg (aspirin content 9 mg/kg), corresponding to a human equivalent dose of 100 mg for 60 kg average body weight. The C_max_ of inhalation administration improved significantly to 44.33 ± 2.60 µg/mL (Table 3), which increased to 1.8 times as compared with oral administration (26.21 ± 4.90 µg/mL). Meanwhile, the AUC_0-∞_ value of the inhalation group (231.88 µg·h/mL) was 1.91 times as the oral group (121.36 µg·h/mL). Besides, the T_max_ of inhalation groups (10 mins) was significantly shorter than that of oral groups (30 mins). Thus, compared to oral administration, the superior value of AUC_0-∞_ and C_max_ demonstrated inhalation administration could dramatically increase the in-vivo bioavailability of ASA. Furthermore, the decreased T_max_ proved that aspirin could be absorbed into the bloodstream rapidly, and revealed faster antiplatelet activity compared with conventional oral products.

### 3.8 In vivo anti-platelet aggregation and safety study

As shown in Fig. S1C, the AA-induced PA of inhale administration was significantly lower than that of oral administration within 10 min. However, the inhibition effect of AA-induced PA tended to be similar after 1 h. In contrast, ADP induced PA by inhale administration was much lower than oral administration within 2 h (Fig. S1D). Thus, the inhibition effect of platelet aggregation via inhalation administration of aspirin inhalable powder is more effectively and rapidly than oral administration.

The safety of continuous oral and intratracheal administration of ASA inhalable powder F6 (9 mg/kg/day) was evaluated through 7-day toxicology study in mice. No mice died after delivering sample F6 within 7 days. Meanwhile, immune cells infiltration caused by particles (including lymphocytes and eosinophils, alveolar septal thickening, alveolar macrophage accumulation and granulomatous lesions) [53] could not be observed in histopathological images (Fig. S1E) of main organs (heart, liver, spleen, lung, kidneys, stomach), indicating that aspirin DPIs had no acute toxicity to mice.

## 4 Conclusion

In this study, aspirin inhalable powders were prepared by ultrasound-intensified anti-solvent crystallization (UIAC). The DSC and XRD analysis exhibited the powder crystallinity prepared by UIAC was reduced, resulting in an improvement in dissolution behavior. The addition of excipients (L150 and leu) could effectively reduce the electrostatic force, as well as improve the flowability and atomization properties. The A549 cytotoxicity, Calu-3 cell monolayer interfacial integrity, and in-vivo safety studies demonstrated the high permeability and safety of aspirin inhalable powder for pulmonary administration. The pharmacokinetics study revealed that inhalation administration could effectively shorten T_max_ and raise bioavailability than oral administration. Inhalation administration inhibited platelet aggregation in rats more strongly and more rapidly than oral administration. In conclusion, ultrasound-intensified anti-solvent crystallization is a reliable method to prepare aspirin inhalable powders for deep pulmonary drug delivery.

## CRediT authorship contribution statement

**Yan Zhao:** Formal analysis, Investigation, Writing – original draft. **Kai Feng:** Writing – original draft, Data curation, Methodology. **Boxin Liu:** Writing – review & editing, Methodology. **Zhihao Zhang:** Methodology, Resources, Writing – original draft, Formal analysis. **Haozhou Huang:** Software, Formal analysis, Data curation. **Mateng Chen:** Methodology, Writing – review & editing. **Qingzhen Zhang:** Data curation, Investigation. **Gang Yang:** Investigation, Methodology, Resources. **Mengxing Lin:** Investigation, Data curation, Methodology, Formal analysis. **Yulong Zhang:** Data curation, Investigation, Methodology, Conceptualization. **Hanhan Li:** Conceptualization, Methodology, Data curation, Formal analysis. **Ning Xue:** Data curation, Software, Project administration. **Kaiqi Shi:** Writing – review & editing, Funding acquisition, Supervision, Writing – original draft. **Qiang Da:** Resources, Writing – review & editing, Funding acquisition, Supervision. **Bin Dong:** Funding acquisition, Methodology, Supervision, Writing – review & editing, Conceptualization.

## Declaration of competing interest

The authors declare that they have no known competing financial interests or personal relationships that could have appeared to influence the work reported in this paper.
