# Supplementary material for: Preparation of aspirin inhalable powder by ultrasound-intensified anti-solvent crystallization for pulmonary drug delivery
Source: Ultrason Sonochem. 2025 Jul 11;120:107464. doi: 10.1016/j.ultsonch.2025.107464 (PMC12296543; doi:10.1016/j.ultsonch.2025.107464)
Supplement: Supplementary Data 1 [file mmc1.docx]

Preparation of aspirin inhalable powder by ultrasound-intensified anti-solvent crystallization for pulmonary drug delivery

Yan Zhao^a,b,#^, Kai Feng^c,#^, Boxin Liu^c^, Zhihao Zhang^c^, Haozhou Huang^d^, Mateng Chen^c^, Qingzhen Zhang^e^, Gang Yang^f^, Mengxing Lin^f^, Yulong Zhang^b,g^, Hanhan Li^b^, Ning Xue^h^, Kaiqi Shi^e,*^, Qiang Da^a,*^, Bin Dong^b,i*^

^a^ The First Affiliated Hospital of Nanjing Medical University, 300 Guangzhou Road, Nanjing 210029, PR China

^b^ Department of Pharmaceutical Engineering, China Pharmaceutical University, Nanjing, 210009, PR China

^c^ State Key Laboratory of Natural Medicines, Key Laboratory of Drug Quality Control and Pharmacovigilance, School of Pharmacy, China Pharmaceutical University, Nanjing 210009, PR China

^d^ Courant Institute of Mathematical Sciences, New York University, USA

^e^ Suzhou Inhal Pharma Co., Ltd, Suzhou, Jiangsu, 215000, PR China

^f^ Eastern Institute for Advanced Study, Eastern Institute of Technology, Ningbo, China

^g^ National Heart & Lung Institute, Faculty of Medicine, Imperial College London, Guy Scadding Building, Cale Street, London, SW3 6LY, United Kingdom

^h^ Department of Computer Science, University of Nottingham Ningbo China, Ningbo, Zhejiang, 31500, China

^i^ Engineering Research Center for Smart Pharmaceutical Manufacturing Technologies, Ministry of Education, China Pharmaceutical University, Nanjing, 210009, PR China

^*^ Corresponding author: skq@inhalpharma.com (Kaiqi Shi); dqph@njmu.edu.cn (Qiang Da); [bin.dong@cpu.edu.cn](mailto:bin.dong@cpu.edu.cn) (Bin Dong)

^#^ Yan Zhao and Kai Feng have contributed equally.

Table S1 Preparation conditions and particle size distribution for all samples

| Sample | Concentration (mg/mL) | Ultrasonic Power (W) | Water/Ethanol (v/v) | Rate of water (mL/min) | D10  (μm) | D50  (μm) | D90  (μm) | D_v_  (μm) |
| --- | --- | --- | --- | --- | --- | --- | --- | --- |
| A1 | 140 | 0 | 10 | 28 | 1.48±0.13 | 5.94±0.95 | 22.31±2.98 | 10.12±0.54 |
| A2 | 140 | 300 | 10 | 28 | 1.26±0.06 | 4.20±0.39 | 11.55±2.46 | 5.69±1.02 |
| A3 | 140 | 600 | 10 | 28 | 1.19±0.04 | 3.82±0.03 | 9.55±0.80 | 4.69±0.21 |
| A4 | 140 | 900 | 10 | 28 | 1.26±0.05 | 4.59±0.31 | 11.78±0.24 | 5.73±0.02 |
| A5 | 140 | 1200 | 10 | 28 | 1.20±0.10 | 4.06±0.59 | 10.61±2.05 | 5.12±0.93 |
| A6 | 130 | 600 | 10 | 28 | 1.29±0.05 | 4.49±0.30 | 11.97±0.92 | 5.75±0.42 |
| A7 | 120 | 600 | 10 | 28 | 1.30±0.08 | 4.57±0.51 | 12.04±0.88 | 5.81±0.51 |
| A8 | 110 | 600 | 10 | 28 | 1.27±0.01 | 4.33±0.03 | 11.74±0.15 | 5.60±0.05 |
| A9 | 100 | 600 | 10 | 28 | 1.36±0.07 | 4.92±0.47 | 14.87±0.51 | 9.32±2.73 |
| A10 | 90 | 600 | 10 | 28 | 1.39±0.13 | 5.14±0.85 | 13.62±1.92 | 6.57±1.04 |
| A11 | 140 | 600 | 20 | 28 | 1.18±0.13 | 3.99±0.64 | 10.33±1.97 | 4.56±0.64 |
| A12 | 130 | 600 | 20 | 28 | 1.27±0.16 | 4.42±0.96 | 11.47±2.68 | 5.59±1.37 |
| A13 | 120 | 600 | 20 | 28 | 1.20±0.07 | 3.99±0.37 | 10.51±1.77 | 5.04±0.73 |
| A14 | 110 | 600 | 20 | 28 | 1.23±0.23 | 4.26±1.30 | 10.17±3.40 | 5.04±1.63 |
| A15 | 100 | 600 | 20 | 28 | 1.17±0.13 | 3.84±0.66 | 9.68±2.85 | 4.74±1.22 |
| A16 | 90 | 600 | 20 | 28 | 1.17±0.07 | 3.83±0.32 | 9.58±1.26 | 4.37±0.17 |
| A17 | 130 | 600 | 5 | 28 | 1.79±0.55 | 6.91±2.19 | 17.08±4.30 | 9.09±3.39 |
| A18 | 120 | 600 | 5 | 28 | 1.38±0.10 | 5.10±0.66 | 13.45±1.66 | 6.52±0.88 |
| A19 | 110 | 600 | 5 | 28 | 1.40±0.33 | 5.28±1.89 | 12.49±5.24 | 6.23±2.43 |
| A20 | 100 | 600 | 5 | 28 | 1.42±0.27 | 5.37±1.64 | 13.09±4.52 | 6.48±2.16 |
| A21 | 90 | 600 | 5 | 28 | 1.37±0.13 | 5.06±0.83 | 13.09±2.10 | 6.33±1.00 |
| A22 | 130 | 600 | 15 | 28 | 1.21±0.04 | 4.06±0.21 | 10.68±0.95 | 5.13±0.40 |
| A23 | 130 | 600 | 10 | 21 | 1.23±0.07 | 4.12±0.35 | 10.53±1.67 | 5.10±0.68 |
| A24 | 130 | 600 | 10 | 14 | 1.65±0.13 | 6.25±0.95 | 14.62±3.22 | 8.06±2.97 |
| A25 | 120 | 600 | 15 | 28 | 1.27±0.15 | 4.35±0.78 | 12.15±1.03 | 5.76±0.52 |
| A26 | 110 | 600 | 15 | 28 | 1.22±0.21 | 4.12±0.53 | 10.89±1.52 | 5.24±1.30 |
| A27 | 100 | 600 | 15 | 28 | 1.32±0.19 | 4.64±0.57 | 13.20±2.01 | 6.59±2.01 |
| A28 | 90 | 600 | 15 | 28 | 1.16±0.11 | 4.90±0.91 | 12.59±1.25 | 6.13±1.06 |
| A29 | 140 | 600 | 10 | 21 | 1.29±0.08 | 4.43±0.76 | 11.90±0.98 | 5.71±1.05 |
| A30 | 120 | 600 | 10 | 21 | 1.21±0.12 | 4.15±0.29 | 10.90±1.23 | 5.27±1.09 |
| A31 | 110 | 600 | 10 | 21 | 1.28±0.21 | 4.38±0.20 | 11.74±1.03 | 5.63±0.98 |
| A32 | 100 | 600 | 10 | 21 | 1.27±0.19 | 4.35±0.31 | 11.90±1.12 | 5.66±0.62 |
| A33 | 90 | 600 | 10 | 21 | 1.36±0.11 | 4.93±0.59 | 14.99±1.06 | 8.06±1.21 |
| A34 | 120 | 600 | 10 | 14 | 1.45±0.10 | 5.52±0.25 | 14.50±1.56 | 8.06±2.97 |
| A35 | 110 | 600 | 10 | 14 | 1.27±0.13 | 4.37±0.67 | 11.58±2.01 | 5.58±0.95 |
| A36 | 100 | 600 | 10 | 14 | 1.49±0.21 | 5.76±1.23 | 14.08±1.25 | 6.92±0.86 |
| A37 | 90 | 600 | 10 | 14 | 1.40±0.16 | 5.23±0.81 | 13.43±1.65 | 6.51±1.03 |

* D10: the 10% of particles in the powders are smaller than this size. D50 50% of the total particles are smaller than this size. D90: 90% of the total particles are smaller than this size. Dv: volume mean diameter, which describes particle size based on their volume.

Table S2 Mixing uniformity values and flowability for mixed formulations

| Sample | ASA (%) | Leu (%) | L150 (%) | D_10_ (μm) | D_50_ (μm) | D_90_ (μm) | D_v_ (μm) | ρ_B_ (g/cm^3^) | ρ_T_ (g/cm^3^) | CI (%) | HR | ASA content uniformity (%) | CV |
| --- | --- | --- | --- | --- | --- | --- | --- | --- | --- | --- | --- | --- | --- |
| A3 | 100 | - | - | 1.19 | 3.82 | 9.55 | 4.69 | 0.04±0.01 | 0.11±0.03 | 62.03±4.30 | 2.63±0.30 | - | - |
| F1 | 69 | 1 | 30 | 1.17 | 3.83 | 10.15 | 4.88 | 0.10±0.03 | 0.17±0.04 | 41.64±0.85 | 1.71±0.02 | 96.52±2.93 | 2.93 |
| F2 | 7 | 3 | 90 | 1.86 | 10.52 | 56.13 | 21.04 | 0.28±0.02 | 0.43±0.03 | 35.27±3.04 | 1.55±0.07 | 95.71±2.64 | 2.64 |
| F3 | 35 | 5 | 60 | 1.30 | 4.54 | 25.54 | 9.96 | 0.17±0.01 | 0.24±0.02 | 27.24±0.64 | 1.37±0.01 | 94.96±1.99 | 1.99 |
| F4 | 9 | 1 | 90 | 1.97 | 12.34 | 63.94 | 24.24 | 0.30±0.02 | 0.44±0.06 | 32.42±4.54 | 1.49±0.10 | 97.84±2.36 | 2.36 |
| F5 | 37 | 3 | 60 | 1.31 | 4.54 | 30.07 | 10.94 | 0.15±0.03 | 0.23±0.04 | 36.64±1.04 | 1.58±0.03 | 95.15±1.25 | 1.25 |
| F6 | 65 | 5 | 30 | 1.16 | 3.80 | 9.85 | 4.74 | 0.10±0.02 | 0.14±0.02 | 27.50±1.48 | 1.38±0.03 | 97.28±2.45 | 2.45 |
| F7 | 39 | 1 | 60 | 1.21 | 4.07 | 12.55 | 6.60 | 0.17±0.01 | 0.26±0.02 | 35.73±1.57 | 1.56±0.04 | 96.12±2.11 | 2.11 |
| F8 | 37 | 3 | 30 | 1.22 | 4.11 | 13.22 | 8.14 | 0.12±0.01 | 0.21±0.02 | 39.47±2.41 | 1.65±0.06 | 93.26±2.30 | 2.30 |
| F9 | 5 | 5 | 90 | 1.85 | 10.89 | 59.91 | 22.44 | 0.31±0.03 | 0.47±0.04 | 33.00±2.13 | 1.49±0.05 | 95.35±1.27 | 1.27 |

Table S3 Temperature measured at five amplifier power

| Time (min) | Temperature (^o^C) | | | | | | | | | | | | | | |
| --- | --- | --- | --- | --- | --- | --- | --- | --- | --- | --- | --- | --- | --- | --- | --- |
|  | 300 W | | | 600 W | | | 900 W | | | 1200 W | | | 1500 W | | |
| 0 | 22 | 22 | 22 | 22 | 22 | 22 | 22 | 22 | 22 | 22 | 22 | 22 | 22 | 22 | 22 |
| 1 | 34 | 30 | 31 | 36 | 36 | 37 | 36 | 36 | 37 | 44 | 42 | 44 | 52 | 49 | 52 |
| 2 | 42 | 38 | 39 | 46 | 45 | 47 | 50 | 49 | 50 | 62 | 58 | 52 | 72 | 70 | 74 |
| 3 | 50 | 44 | 47 | 55 | 54 | 55 | 61 | 60 | 60 | 76 | 71 | 74 | 87 | 83 | 88 |
| 4 | 57 | 52 | 54 | 62 | 62 | 63 | 71 | 70 | 70 | 84 | 82 | 84 | 96 | 91 | 97 |
| 5 | 62 | 58 | 60 | 70 | 68 | 69 | 79 | 77 | 77 | 92 | 90 | 90 | 100 | 96 | 100 |
| 6 | 67 | 64 | 65 | 75 | 74 | 74 | 84 | 82 | 82 | 96 | 94 | 94 | 100 | 100 | 100 |
| 7 | 72 | 68 | 70 | 80 | 78 | 79 | 89 | 87 | 88 | 98 | 96 | 97 | 100 | 100 | 100 |
| 8 | 75 | 73 | 75 | 83 | 82 | 82 | 92 | 90 | 90 | 100 | 98 | 99 | 100 | 100 | 100 |
| 9 | 80 | 76 | 78 | 86 | 85 | 85 | 94 | 92 | 93 | 100 | 100 | 100 | 100 | 100 | 100 |
| 10 | 82 | 79 | 81 | 89 | 87 | 88 | 95 | 94 | 94 | 100 | 100 | 100 | 100 | 100 | 100 |

Table S4 Applied ultrasound power, temperature difference, and calculated calorimetric power at five amplifier power.

| Applied power  (W) | *ΔT* after 4 min  (°C) | Calorimetric power  (W) |
| --- | --- | --- |
| 1500 | 72.7 | 3.05 |
| 1200 | 61.3 | 2.58 |
| 900 | 48.3 | 2.03 |
| 600 | 40.3 | 1.69 |
| 300 | 32.3 | 1.36 |

Table S5 Composition of the Simulated Lung Fluids

| **Composition (g·L^-1^)** | **PBS** | **GMB** | **ALF** |
| --- | --- | --- | --- |
| NaCl | 8.77 | 6.779 | 3.21 |
| Na_2_HPO_4_ | 1.28 |  | 0.071 |
| NaHCO_3_ |  | 2.268 |  |
| Trisodium citrate dihydrate |  | 0.055 | 0.077 |
| NH_4_Cl |  | 0.535 |  |
| Glycine |  | 0.375 | 0.059 |
| NaH_2_PO_4_ |  | 1.872 |  |
| L-cysteine |  | 0.121 |  |
| NaOH |  |  | 6.0 |
| Citric acid |  |  | 20.8 |
| CaCl_2_·2H_2_O |  | 0.026 | 0.128 |
| Na_2_SO_4_ |  |  | 0.039 |
| MgCl_2_·6H_2_O |  |  | 0.05 |
| Disodium tartrate |  |  | 0.09 |
| Sodium lactate |  |  | 0.085 |
| Sodium pyruvate |  |  | 0.172 |
| Properties |  |  |  |
| pH |  | 7.3 ± 0.1 | 4.5 ± 0.1 |
| Ionic strength  (mol·L^-1^) |  | 0.17 | 0.34 |

Table S6 Bulk densities, Tap densities, CI, and HR of 15 aspirin dry particle samples

(mean ± SD，n=3)

| Sample | D_50_ (μm) | ρ_B_ (g/cm^3^) | ρ_T_ (g/cm^3^) | CI (%) | HR |
| --- | --- | --- | --- | --- | --- |
| A2 | 4.20±0.39 | 0.0206±0.0008 | 0.0480±0.0007 | 57.14±3.2% | 2.33±0.2 |
| A3 | 3.82±0.03 | 0.0413±0.0018 | 0.1088±0.029 | 62.03±4.3% | 2.63±0.3 |
| A4 | 4.59±0.31 | 0.0366±0.0023 | 0.0976±0.027 | 62.50±5.1% | 2.67±0.3 |
| A5 | 4.06±0.59 | 0.0279±0.0026 | 0.0788±0.028 | 64.58±6.8% | 2.82±0.4 |
| A6 | 4.49±0.30 | 0.0370±0.0013 | 0.0867±0.018 | 57.33±3.6% | 2.34±0.3 |
| A7 | 4.57±0.51 | 0.0321±0.0011 | 0.0701±0.016 | 54.21±4.1% | 2.18±0.5 |
| A8 | 4.33±0.03 | 0 .0313±0.0009 | 0.0693±0.0013 | 54.83±3.2% | 2.21±0.4 |
| A11 | 3.99±0.64 | 0.0312±0.0015 | 0.0714±0.0022 | 56.30±5.2% | 2.29±0.6 |
| A12 | 4.42±0.96 | 0.0323±0.0019 | 0.0756±0.0025 | 54.25±6.3% | 2.19±0.7 |
| A13 | 3.99±0.37 | 0.0329±0.0016 | 0.0732±0.0019 | 55.05±4.8% | 2.22±0.5 |
| A14 | 4.26±1.30 | 0.0290±0.0010 | 0.0654±0.0016 | 55.60±3.9% | 2.25±0.4 |
| A15 | 3.84±0.66 | 0.0379±0.0012 | 0.0831±0.021 | 54.46±4.0% | 2.20±0.4 |
| A16 | 3.83±0.32 | 0.0425±0.0015 | 0.1057±0.023 | 59.82±3.6% | 2.49±0.3 |
| A22 | 4.06±0.21 | 0.0338±0.0023 | 0.0898±0.0030 | 62.32±5.9% | 2.65±0.3 |
| A23 | 4.12±0.35 | 0.0361±0.0014 | 0.0959±0.0031 | 62.33±4.7% | 2.65±0.3 |

Table S7 Mixing uniformity values and flowability of mixed formulations

| Sample | *D_50_*  (μm) | *D_v_*  (μm) | *ρ_B_*  (g/cm^3^) | *ρ_T_*  (g/cm^3^) | CI  (%) | HR | ASA content uniformity (%) | CV |
| --- | --- | --- | --- | --- | --- | --- | --- | --- |
| A3 | 3.82 | 4.69 | 0.04±0.01 | 0.11±0.03 | 62.03±4.30 | 2.63±0.30 | -- | -- |
| F1 | 3.83 | 4.88 | 0.10±0.03 | 0.17±0.04 | 41.64±0.85 | 1.71±0.02 | 96.52±2.93 | 2.93 |
| F2 | 10.52 | 21.04 | 0.28±0.02 | 0.43±0.03 | 35.27±3.04 | 1.55±0.07 | 95.71±2.64 | 2.64 |
| F3 | 4.54 | 9.96 | 0.17±0.01 | 0.24±0.02 | 27.24±0.64 | 1.37±0.01 | 94.96±1.99 | 1.99 |
| F4 | 12.34 | 24.24 | 0.30±0.02 | 0.44±0.06 | 32.42±4.54 | 1.49±0.10 | 97.84±2.36 | 2.36 |
| F5 | 4.54 | 10.94 | 0.15±0.03 | 0.23±0.04 | 36.64±1.04 | 1.58±0.03 | 95.15±1.25 | 1.25 |
| F6 | 3.80 | 4.74 | 0.10±0.02 | 0.14±0.02 | 27.50±1.48 | 1.38±0.03 | 97.28±2.45 | 2.45 |
| F7 | 4.07 | 6.60 | 0.17±0.01 | 0.26±0.02 | 35.73±1.57 | 1.56±0.04 | 96.12±2.11 | 2.11 |
| F8 | 4.11 | 8.14 | 0.12±0.01 | 0.21±0.02 | 39.47±2.41 | 1.65±0.06 | 93.26±2.30 | 2.30 |
| F9 | 10.89 | 22.44 | 0.31±0.03 | 0.47±0.04 | 33.00±2.13 | 1.49±0.05 | 95.35±1.27 | 1.27 |


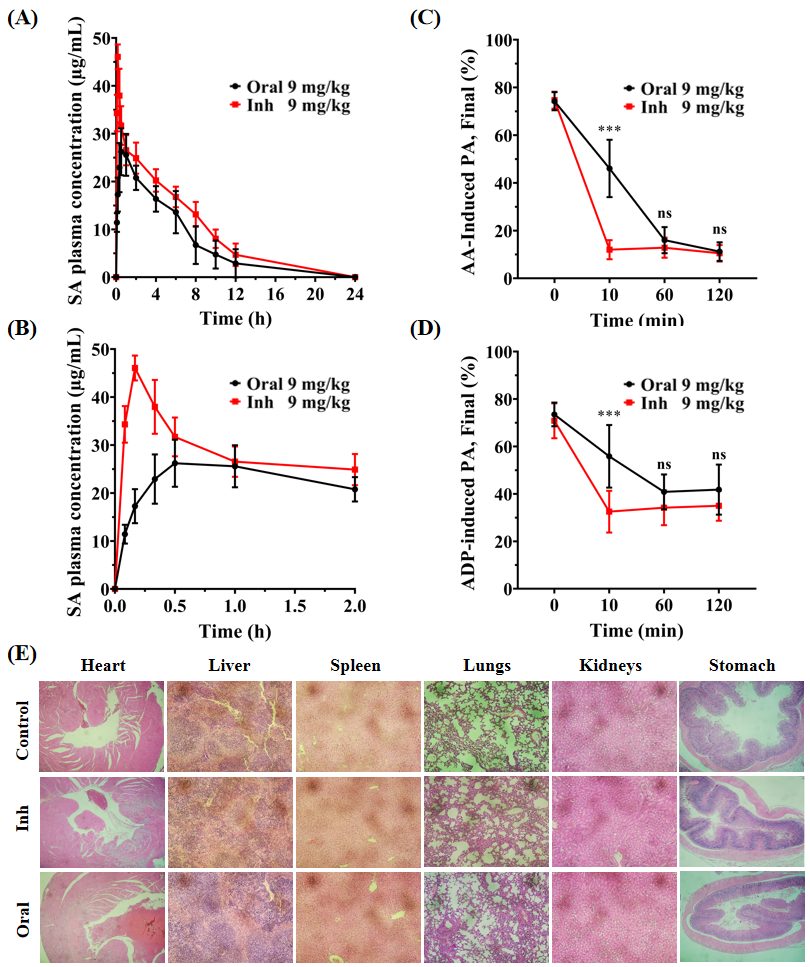


Figure S1. (A-B) SA plasma concentration after inhale and oral administration; (C) Arachidonic acid (AA)-induced platelet aggregation; (D) Adenosine diphosphate (ADP)-induced platelet aggregation; (E) H&E staining of main tissues (including heart, liver, spleen, lung, kidneys, stomach).
